# Supplementary figures and images for: A parasitic fungus employs mutated eIF4A to survive on rocaglate-synthesizing Aglaia plants
Source: eLife. 2023 Feb 28;12:e81302. doi: 10.7554/eLife.81302 (PMC9977294; doi:10.7554/eLife.81302)

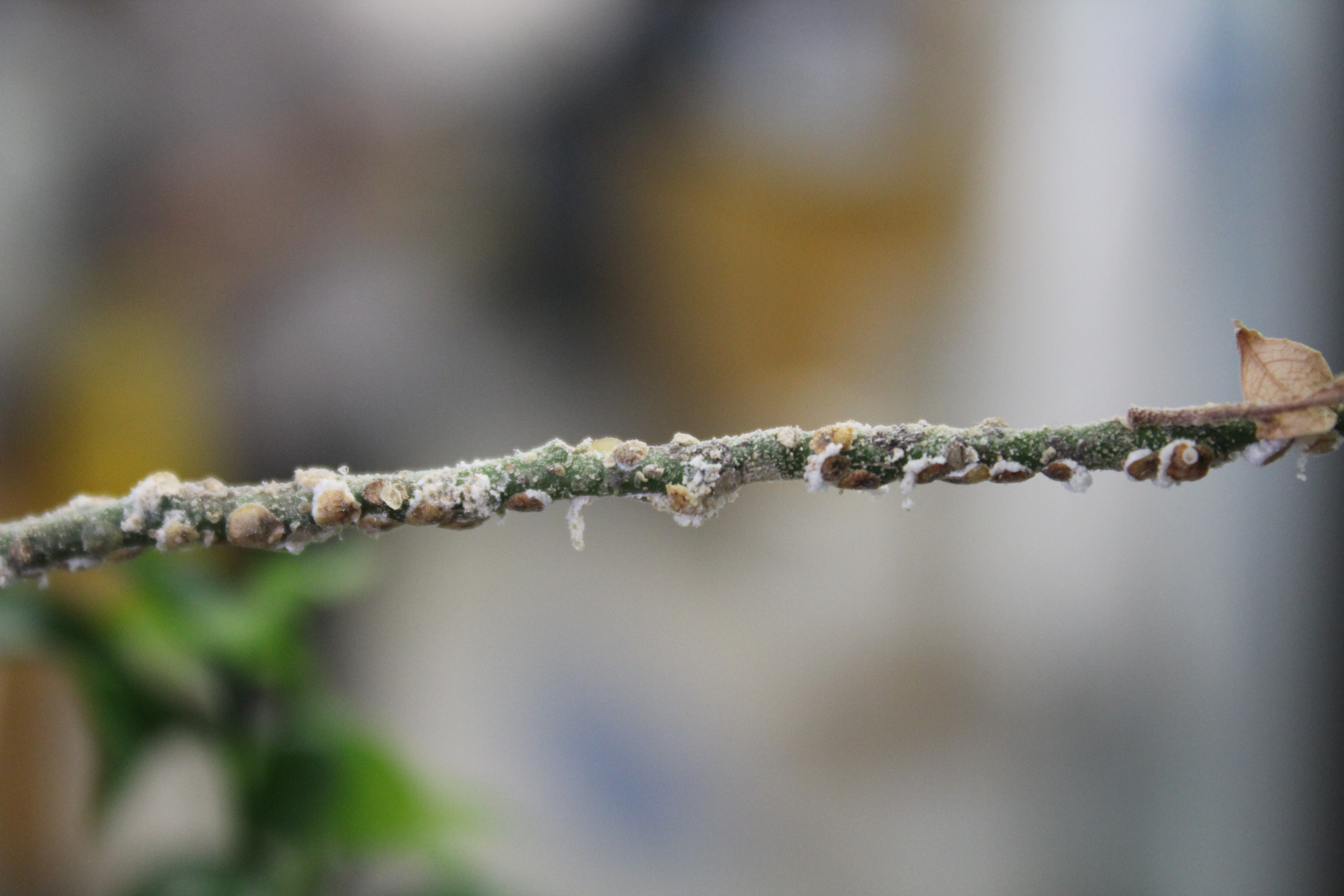

Supplement: Figure 1—source data 1. [file elife-81302-fig1-data1.zip › Figure 1-source data 1/Figure 1A-source data 1.JPG]

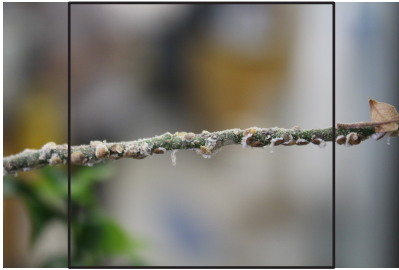

Supplement: Figure 1—source data 1. [file elife-81302-fig1-data1.zip › Figure 1-source data 1/Figure 1A.pdf]

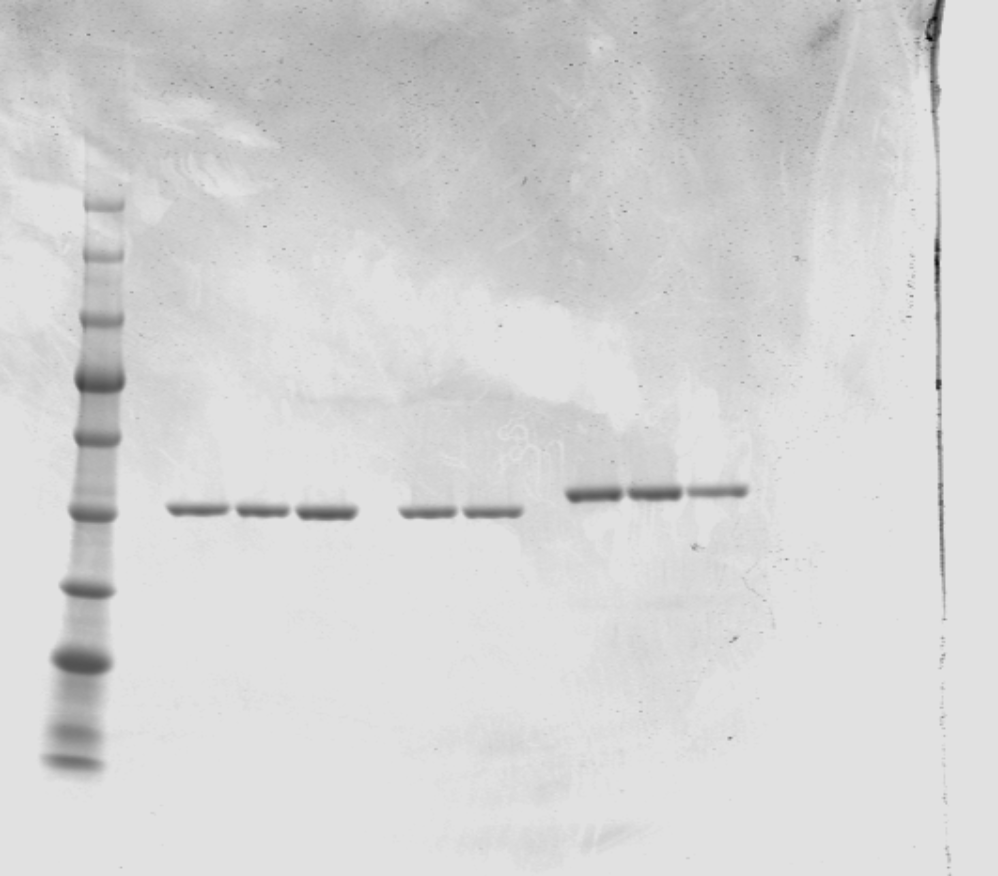

Supplement: Figure 2—figure supplement 1—source data 1. [file elife-81302-fig2-figsupp1-data1.zip › Figure 2-figure supplement 1-source data 1/Figure 2-figure supplement 1B-source data 1.tif]

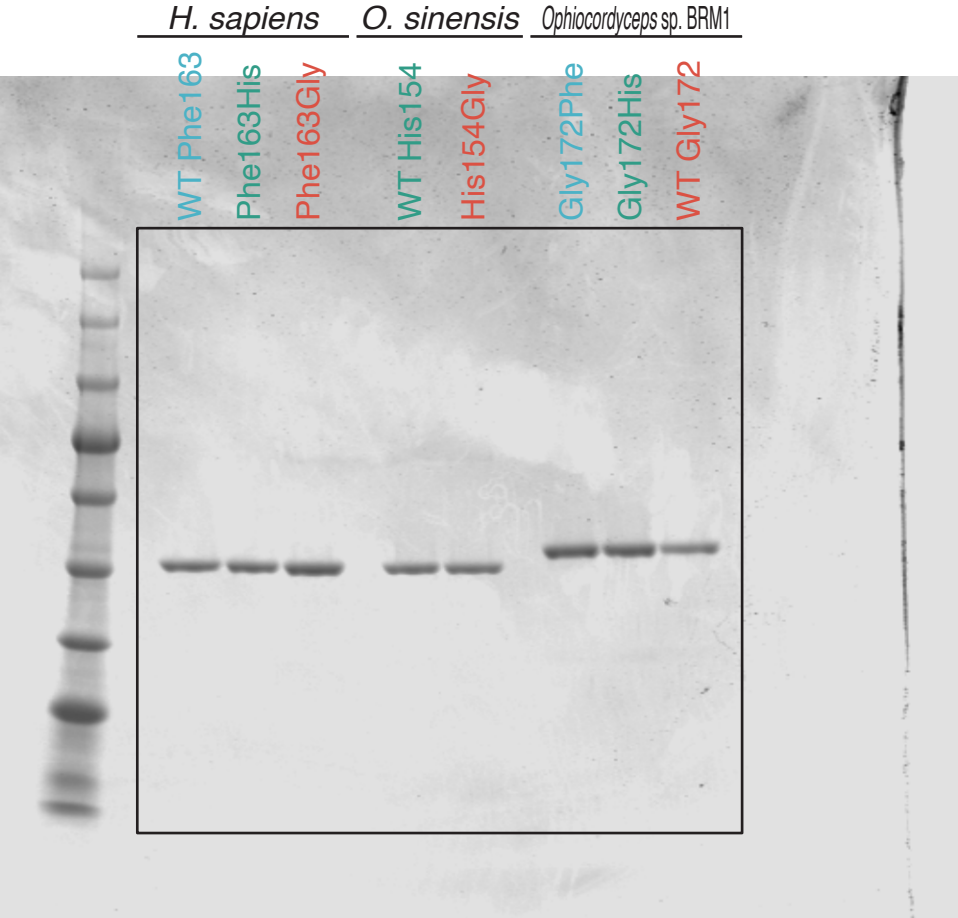

Supplement: Figure 2—figure supplement 1—source data 1. [file elife-81302-fig2-figsupp1-data1.zip › Figure 2-figure supplement 1-source data 1/Figure 2-figure supplement 1B.pdf]

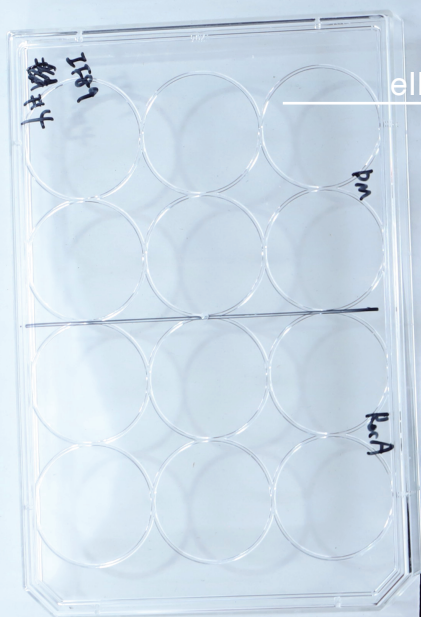

eIF4A recombined

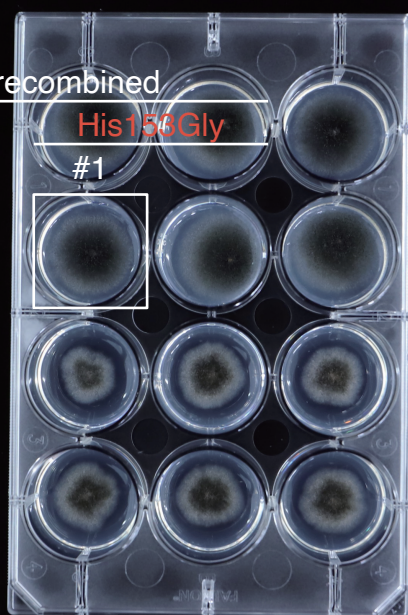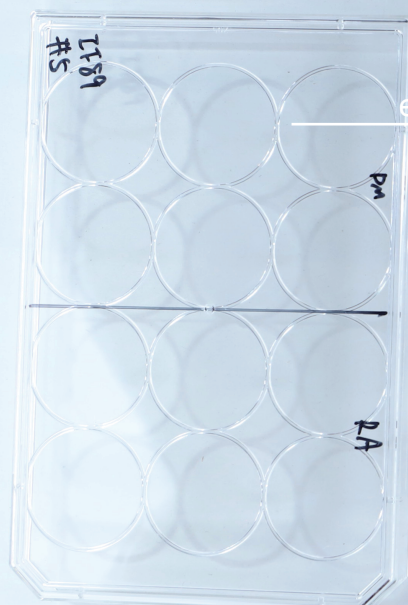

eIF4A recombined

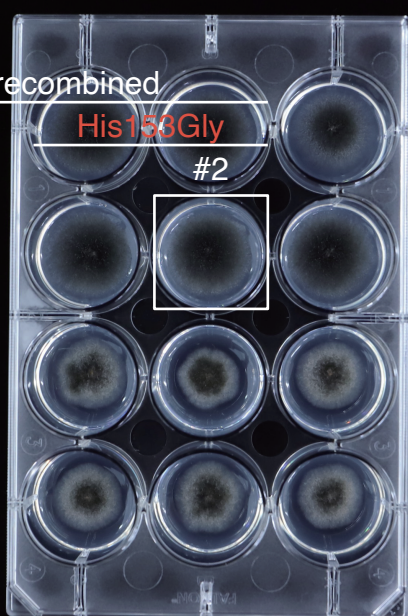

Supplement: Figure 3—figure supplement 1—source data 1. [file elife-81302-fig3-figsupp1-data1.zip › Figure 3-figure supplement 1-source data 1/Figure 3-figure supplement 1C_His153Gly.pdf]

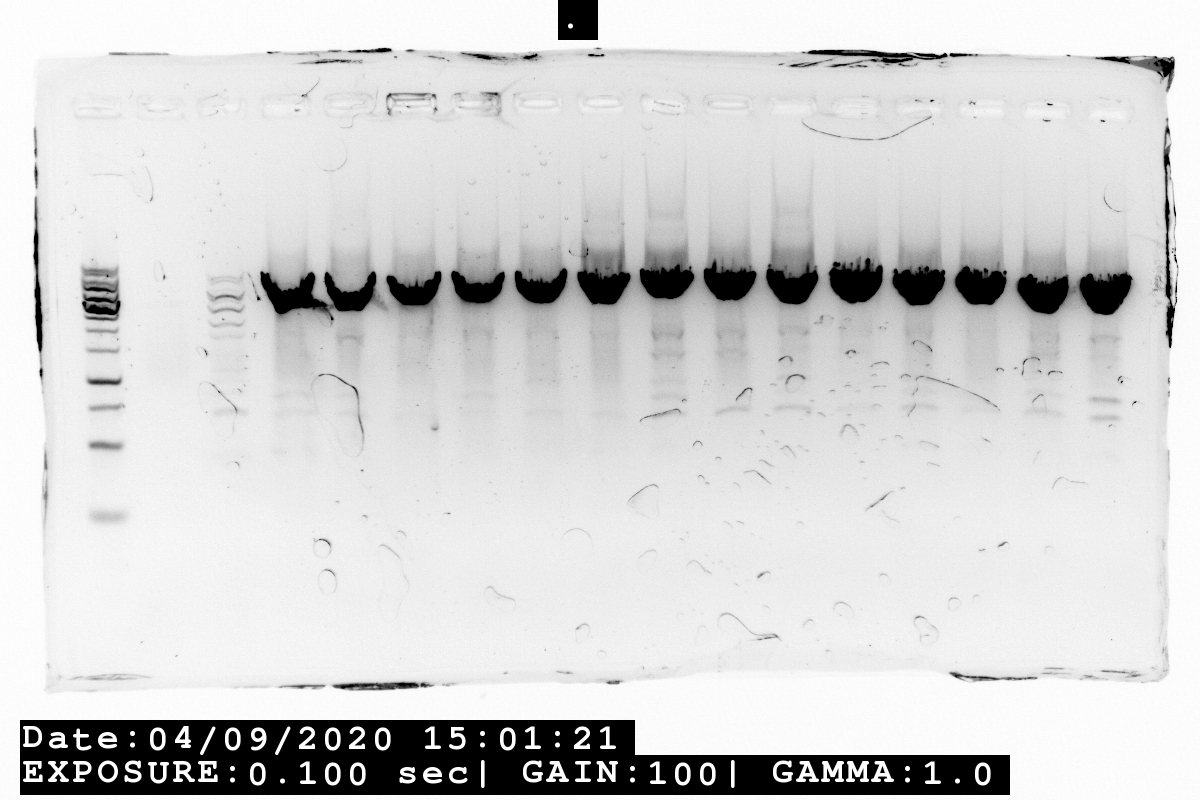

Supplement: Figure 3—figure supplement 1—source data 1. [file elife-81302-fig3-figsupp1-data1.zip › Figure 3-figure supplement 1-source data 1/Figure 3-figure supplement 1B-source data 2.jpeg]

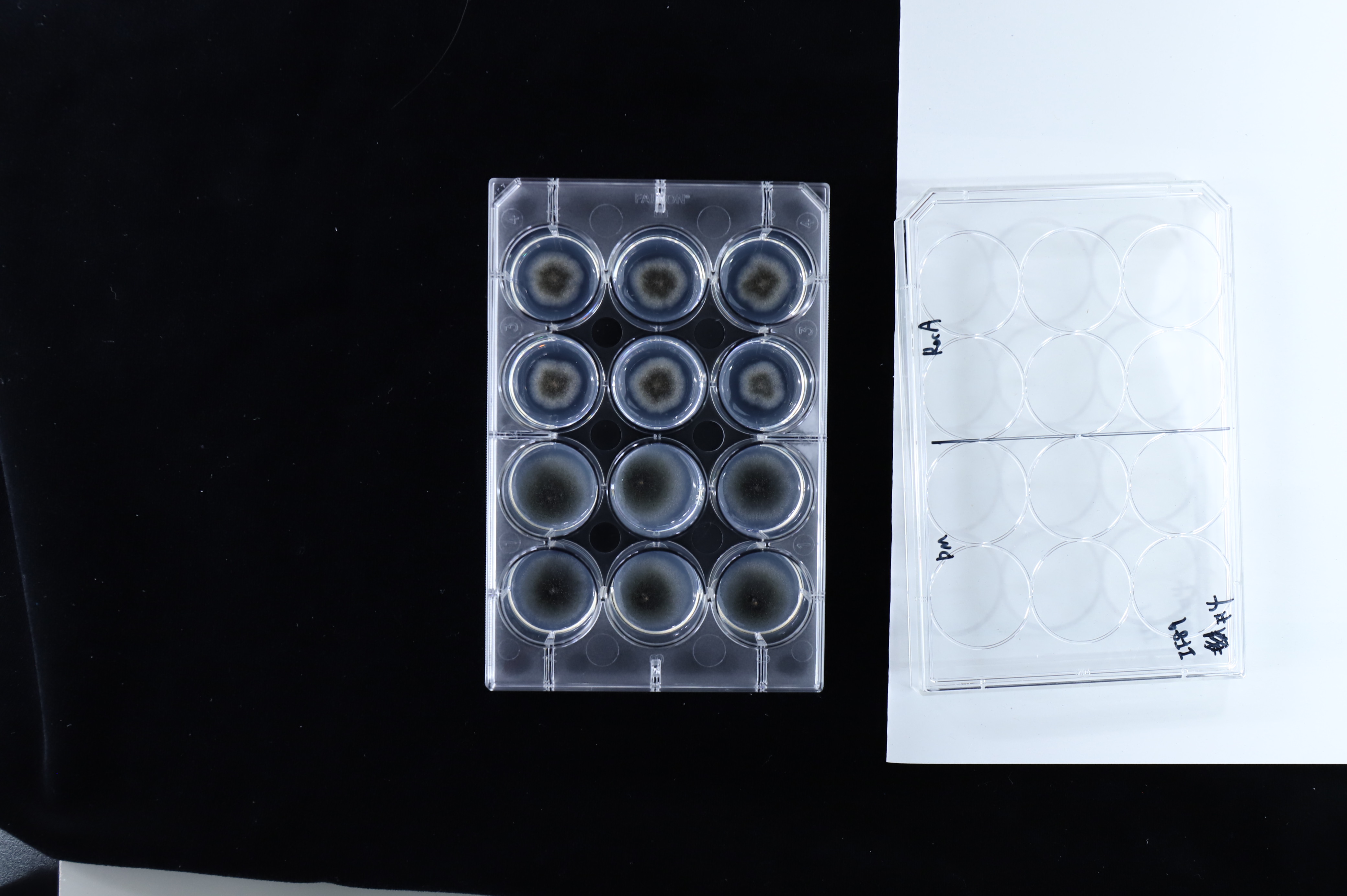

Supplement: Figure 3—figure supplement 1—source data 1. [file elife-81302-fig3-figsupp1-data1.zip › Figure 3-figure supplement 1-source data 1/Figure 3-figure supplement 1C-source data 4.JPG]

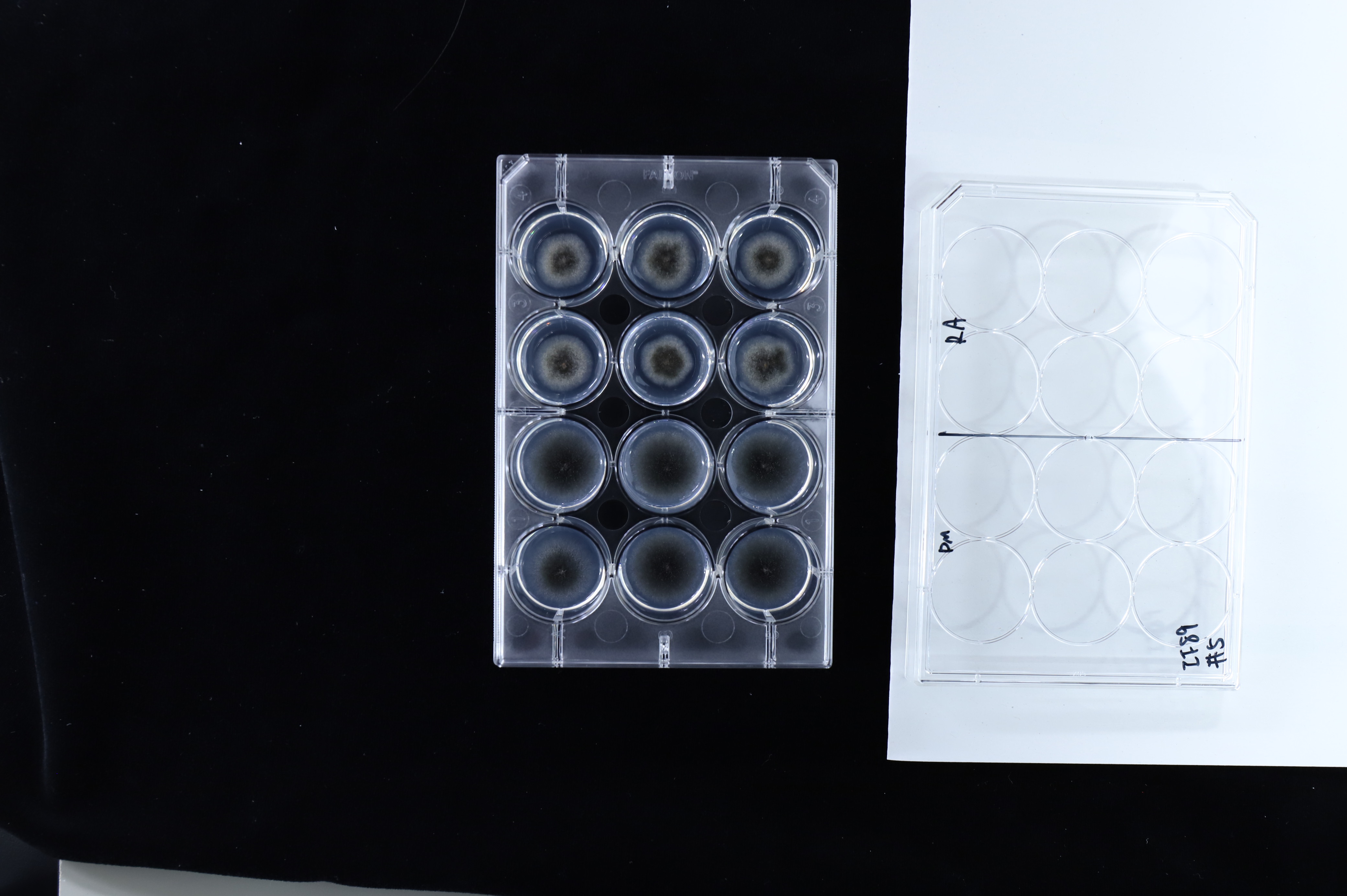

Supplement: Figure 3—figure supplement 1—source data 1. [file elife-81302-fig3-figsupp1-data1.zip › Figure 3-figure supplement 1-source data 1/Figure 3-figure supplement 1C-source data 5.JPG]

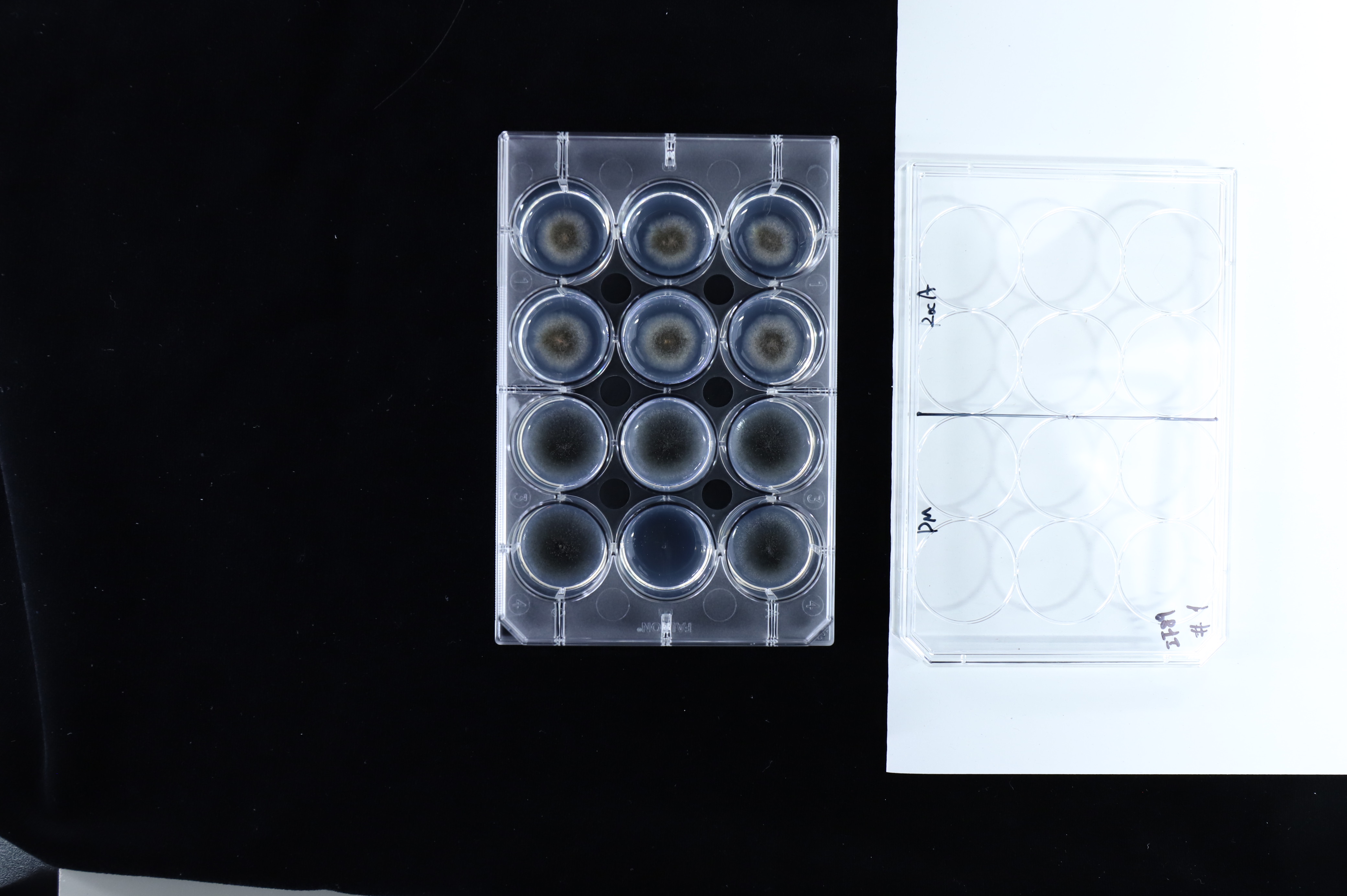

Supplement: Figure 3—figure supplement 1—source data 1. [file elife-81302-fig3-figsupp1-data1.zip › Figure 3-figure supplement 1-source data 1/Figure 3-figure supplement 1C-source data 1.JPG]

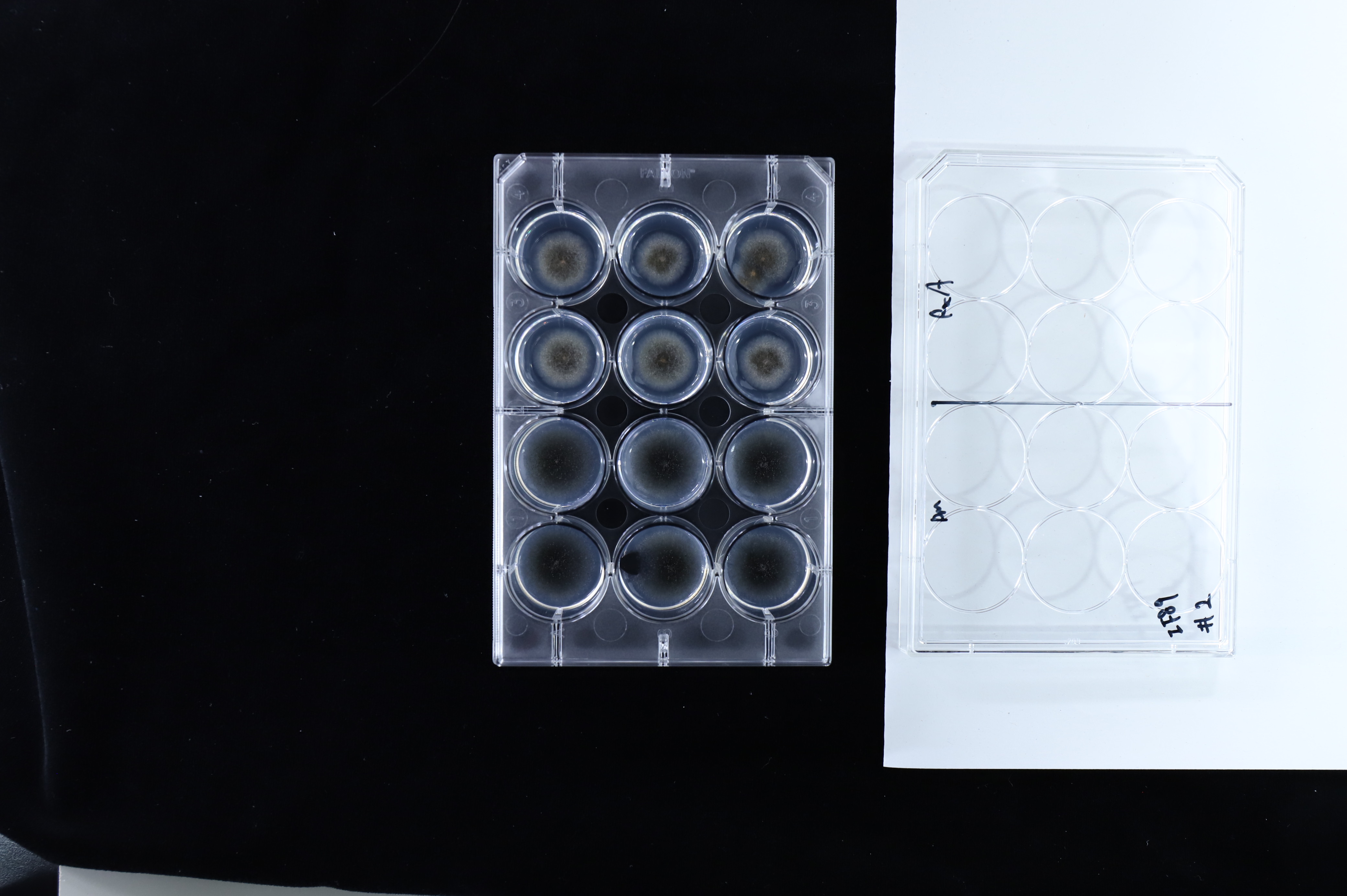

Supplement: Figure 3—figure supplement 1—source data 1. [file elife-81302-fig3-figsupp1-data1.zip › Figure 3-figure supplement 1-source data 1/Figure 3-figure supplement 1C-source data 2.JPG]

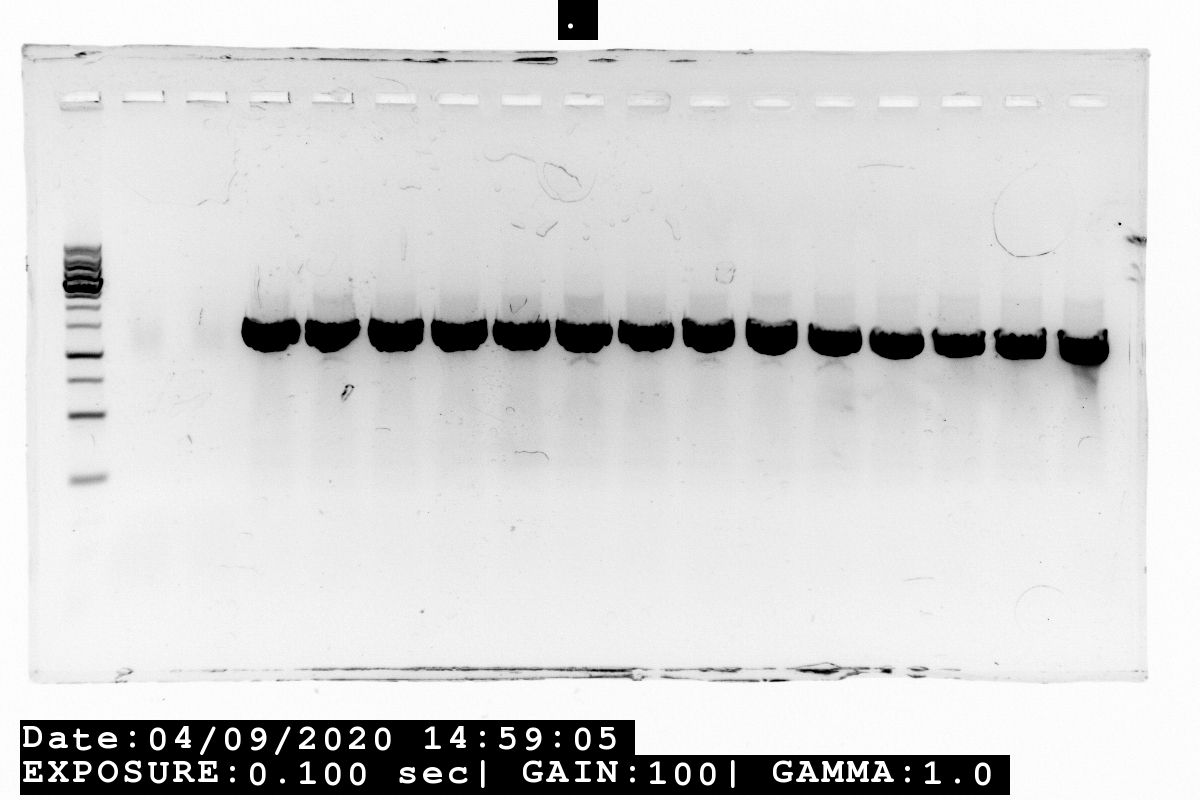

Supplement: Figure 3—figure supplement 1—source data 1. [file elife-81302-fig3-figsupp1-data1.zip › Figure 3-figure supplement 1-source data 1/Figure 3-figure supplement 1B-source data 3.jpeg]

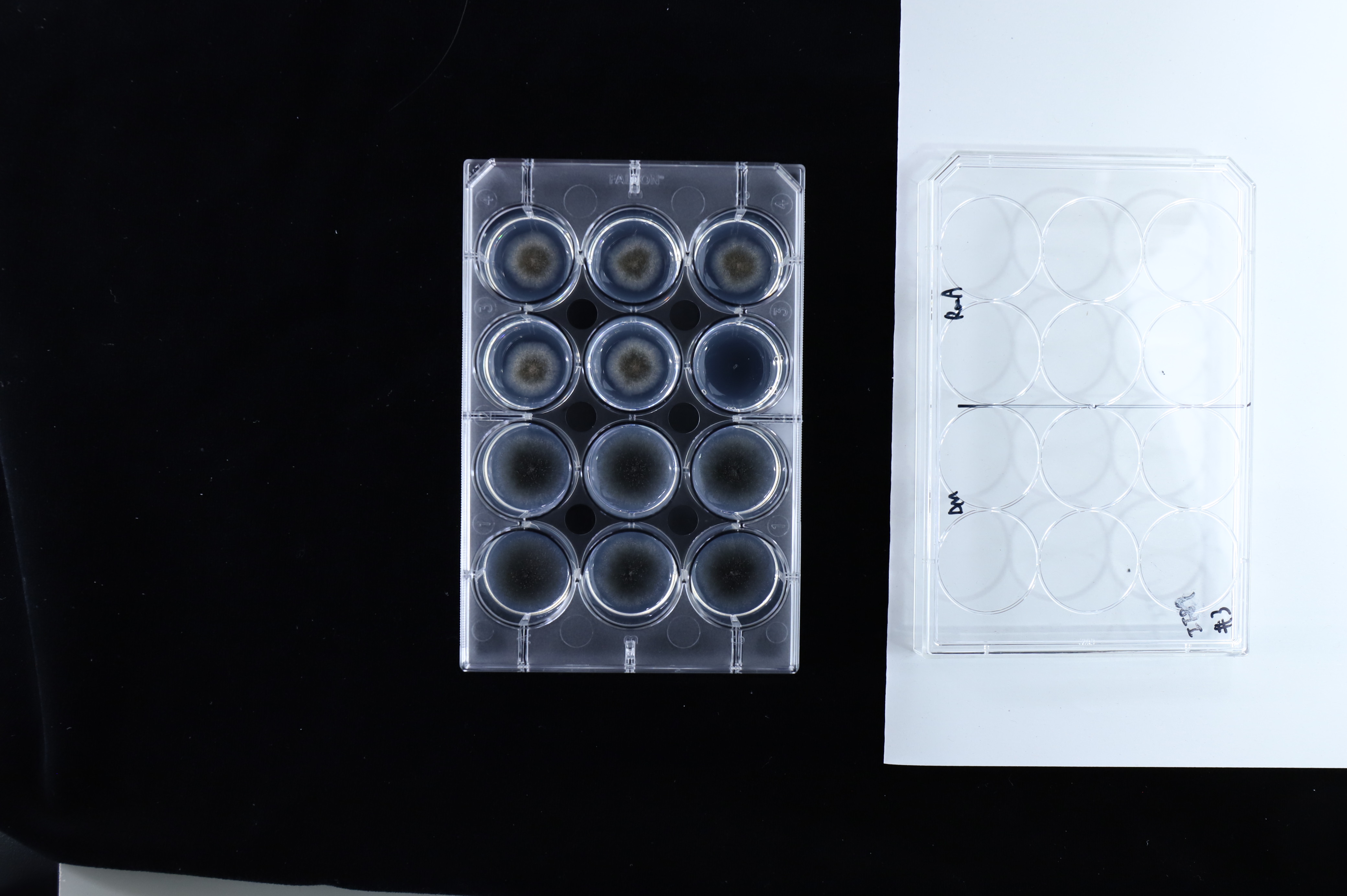

Supplement: Figure 3—figure supplement 1—source data 1. [file elife-81302-fig3-figsupp1-data1.zip › Figure 3-figure supplement 1-source data 1/Figure 3-figure supplement 1C-source data 3.JPG]

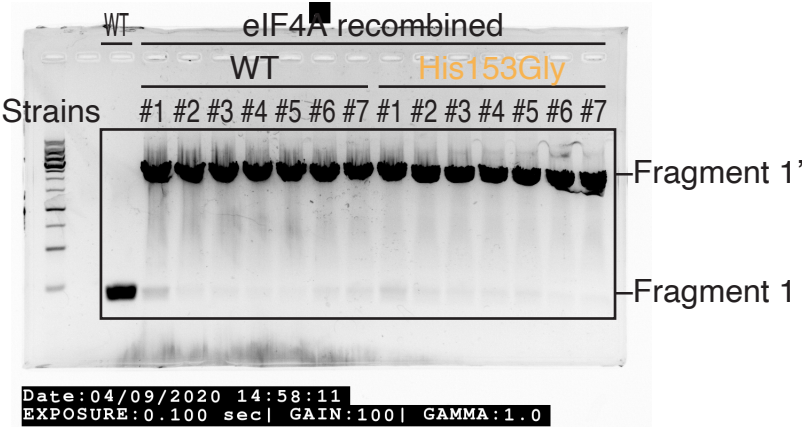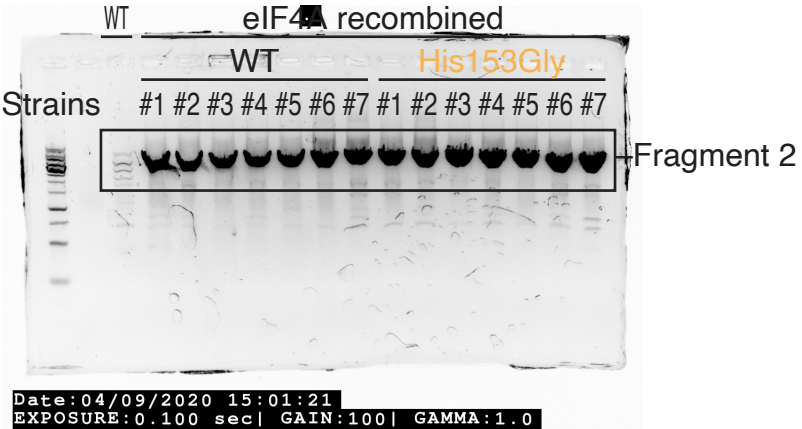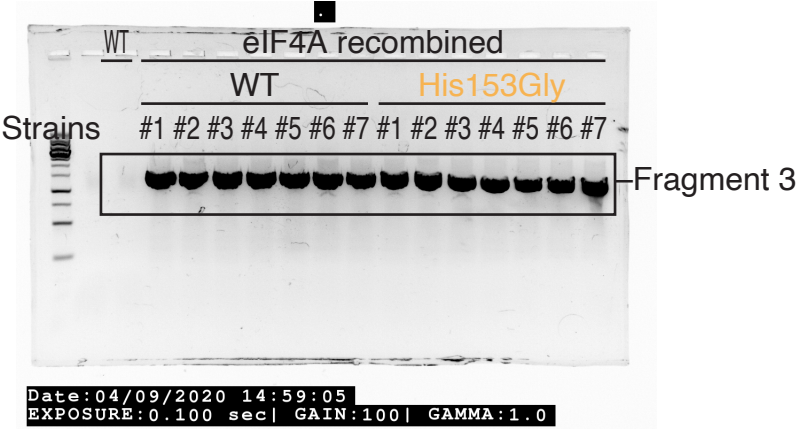

Supplement: Figure 3—figure supplement 1—source data 1. [file elife-81302-fig3-figsupp1-data1.zip › Figure 3-figure supplement 1-source data 1/Figure 3-figure supplement 1B.pdf]

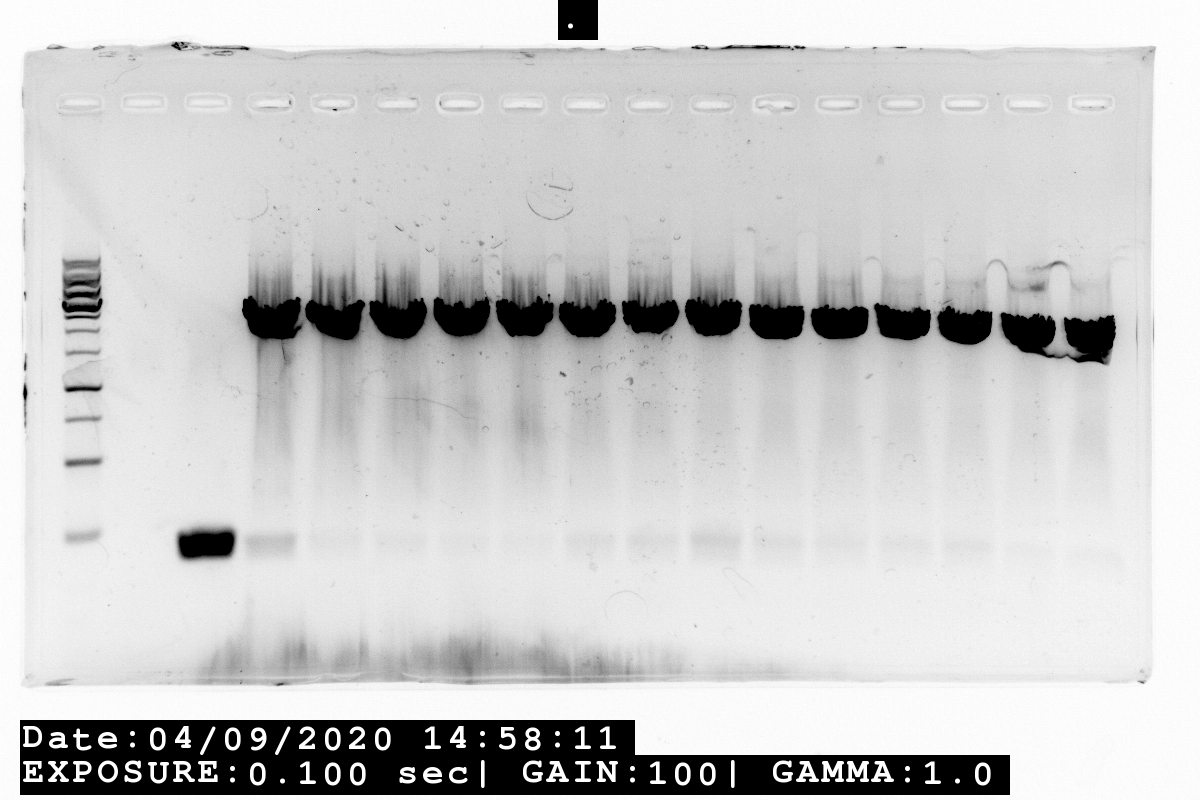

Supplement: Figure 3—figure supplement 1—source data 1. [file elife-81302-fig3-figsupp1-data1.zip › Figure 3-figure supplement 1-source data 1/Figure 3-figure supplement 1B-source data 1.jpeg]

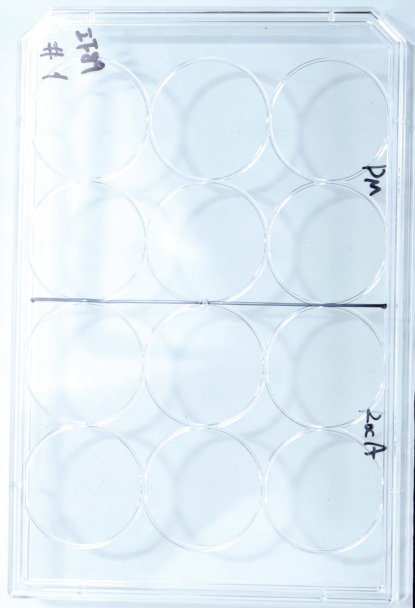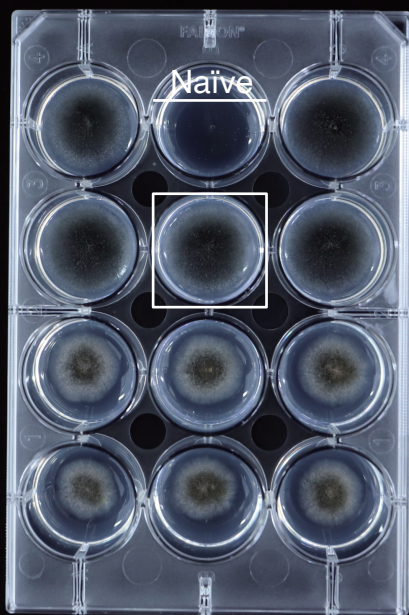

Supplement: Figure 3—figure supplement 1—source data 1. [file elife-81302-fig3-figsupp1-data1.zip › Figure 3-figure supplement 1-source data 1/Figure 3-figure supplement 1C_naive.pdf]

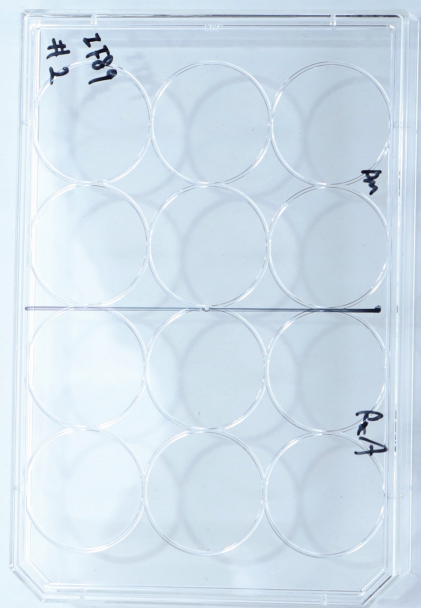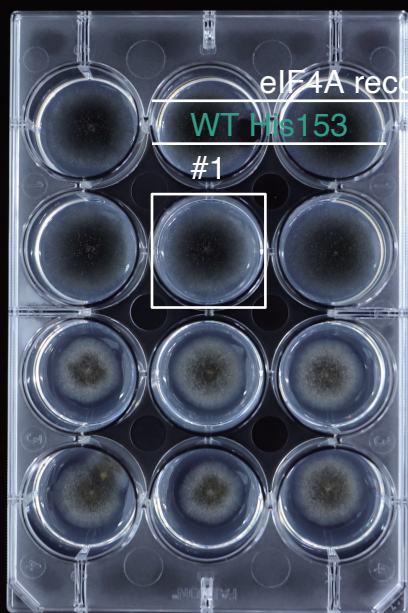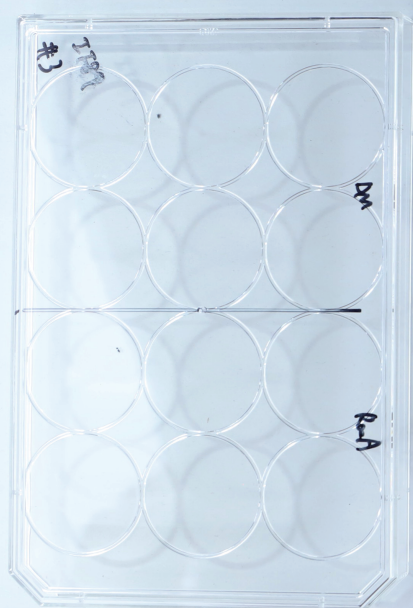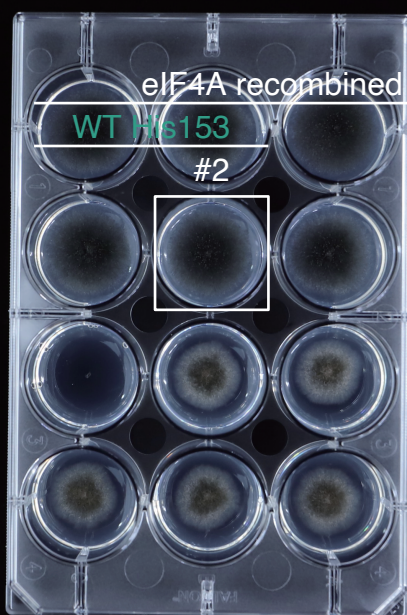

Supplement: Figure 3—figure supplement 1—source data 1. [file elife-81302-fig3-figsupp1-data1.zip › Figure 3-figure supplement 1-source data 1/Figure 3-figure supplement 1C_WT.pdf]

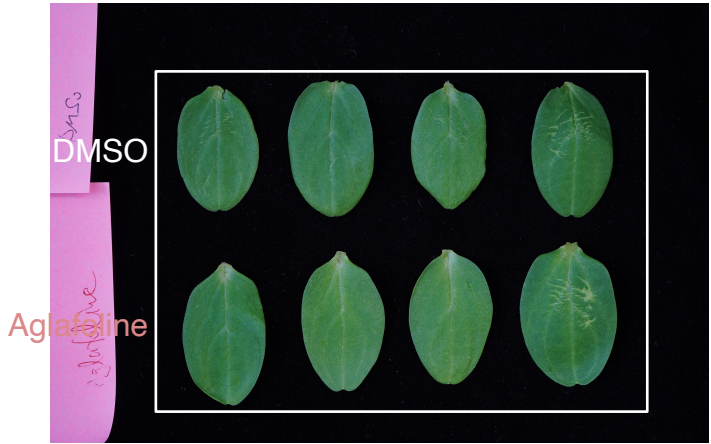

Supplement: Figure 4—figure supplement 1—source data 1. [file elife-81302-fig4-figsupp1-data1.zip › Figure 4-figure supplement 1-source data 1/Figure 4-figure supplement 1A.pdf]

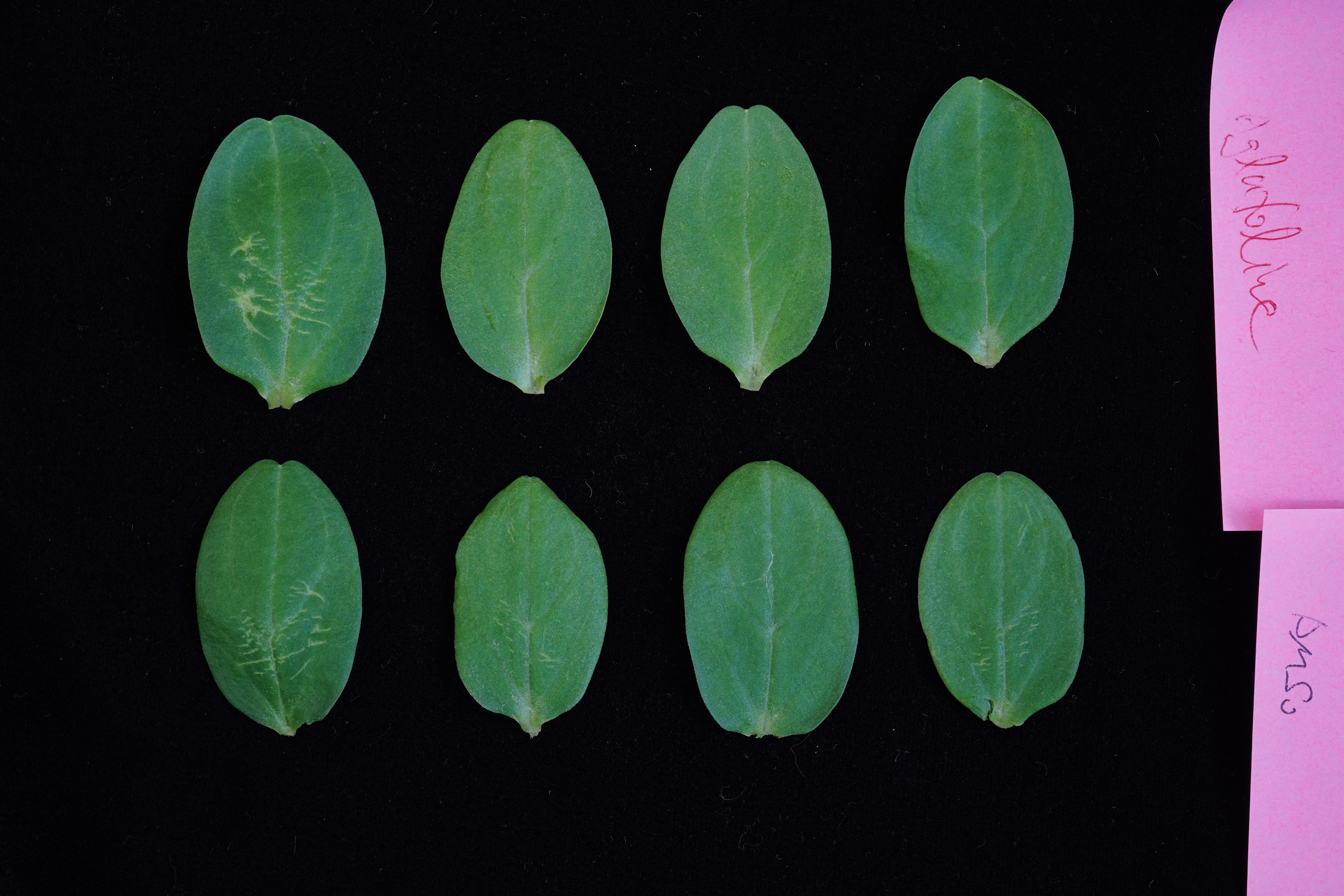

Supplement: Figure 4—figure supplement 1—source data 1. [file elife-81302-fig4-figsupp1-data1.zip › Figure 4-figure supplement 1-source data 1/Figure 4-figure supplement 1A-source data 1.JPG]
